# Underreporting of Congenital Syphilis as a Cause of Fetal and Infant Deaths in Northeastern Brazil

**DOI:** 10.1371/journal.pone.0167255

**Published:** 2016-12-12

**Authors:** Ana Rita Paulo Cardoso, Maria Alix Leite Araújo, Roumayne Fernandes Vieira Andrade, Valéria Saraceni, Angelica E. Miranda, Maria Inês Costa Dourado

**Affiliations:** 1 Collective Health Post Graduation Program. University of Fortaleza, Fortaleza, Brazil; 2 Rio de Janeiro Municipal Secretary of Health, Rio de Janeiro, Brazil; 3 Department of Social Medicine. Federal University of Espírito Santo, Vitória, Brazil; 4 Institute of Collective Heath. Federal University of Bahia, Salvador, Brazil; Centre Hospitalier Universitaire Vaudois, FRANCE

## Abstract

**Introduction:**

Of all syphilis-related pregnancy outcomes, fetal death is certainly the most common one, being directly related to the availability, accessibility and quality of prenatal care. The present study aimed to analyze the underreporting of fetal and infant deaths and other maternal factors associated with congenital syphilis (CS) death.

**Methods:**

This cross-sectional study integrated data of infants that were diagnosed and/or died of CS from the *Sistema de Informação de Agravos de Notificação–Sinan* (Notifiable Diseases Information System) and the *Sistema de Informação de Mortalidade–SIM* (Mortality Information System) in Fortaleza, Northeastern Brasil to identify unreported cases of congenital syphilis. We assessed data during the period from 2007 to 2013.

**Results:**

The underreporting of CS as a cause of fetal or infant death increased from 41 to 415 cases (90.1%) during 2007–2013. Exactly 3,209 cases of CS were identified in Sinan and 6,578 deaths in SIM. After database linkage, we identified 382 cases that were reported in the SIM and SINAN databases consisting of 309 fetal deaths and 73 infant deaths related to CS. From the children notified at Sinan that born alive, 3.0% (78/2,542) died; Out of these, 39 (50.0%) were early and 25 (32.1%) were late neonatal deaths. The proportion of death by CS increased from 0.62 to 5.8 from 2007 to 2013. At logistic regression, the variable that maintained statistical significance with fetal and infant death outcomes was the presence of CS signs and/or symptoms at birth (OR = 3.20; IC 95% 1.54–6.62; p = 0.002).

**Conclusions:**

Neonatal and Infant deaths following CS-associated live births are underreported in Northeastern Brazil. Data base linkage identified unreported fetal and neonatal deaths due to CS leading to an increased awareness of fetal/infant mortality due to this infection.

## Introduction

Syphilis–a sexually transmitted infection–can pass from an infected pregnant woman (GS) to her unborn child–congenital syphilis (CS)–resulting in adverse pregnancy outcomes such as miscarriage, stillbirth, neonatal death, prematurity, and low birth weight [1]. CS is still a major public health problem. However, it can be prevented and controlled, as long as infected pregnant women are adequately diagnosed and promptly treated during prenatal care. Of all syphilis-related pregnancy outcomes, fetal death is certainly the most common one [2,3], being directly related to the quality of prenatal care [4].

The World Health Organization/Pan American Health Organization (WHO/PAHO) committed to reducing its incidence to 0.5 cases per 1,000 live births (LB) by the year 2015. To do so, they proposed to increase to more than 95% the number of countries in the region that have information systems for monitoring and evaluation of progress towards its elimination and support decision-making. Thus, these organizations propose the implementation of a national system for surveillance, monitoring and evaluation of some indicators linked to the incidence of CS [5].

In Brazil, epidemiologic surveillance systems have already been implemented, and they include the monitoring of the main causes of morbidity and mortality such as maternal syphilis and CS. The main surveillance systems in Brazil are the *Sistema de Informação de Agravos de Notificação–Sinan* (Notifiable Diseases Information System) and the *Sistema de Informação de Mortalidade*–SIM (Mortality Information System). However, quality flaws in the registration of CS cases and deaths hinder a reliable analysis of the problem [5], a situation that is also identified in other countries of Latin American and Caribbean [6]. Therefore, despite the magnitude of the problem, morbidity and mortality rates from this disease remain underreported [2,4].

The control of CS and GS is a goal of public health policies in Brazil. However, the incidence rate of CS has been increasing over the past years. In 2013, the incidence rate was 4.7 cases per 1,000 LB, with a higher incidence in Northeastern Brazil, with 5.3 cases per 1,000 LB [7]. A total of 979 cases of CS have been reported in the state of Ceará in 2013, with an incidence rate of 7.7 per 1,000 LB [8]. The city of Fortaleza, the capital of the state of Ceará, holds the vast majority of cases reported in the state [8].

Given the above, the present study aimed to analyze the underreporting of fetal and infant deaths from CS and other maternal factors associated with CS death in a large city in Northeastern Brazil.

## Methods

This was a cross-sectional study conducted in Fortaleza, city with 2.5 million inhabitants located in Northeastern Brazil. The Brazilian Health Ministry developed SIM and Sinan, computer systems for collecting data about mortality and complains of compulsory reporting within Brazil territory. The data obtained through these systems are normally typed in the event occurrence institution, and sent to the Local Health Department, where they are processed, analyzed and transferred to the state database and later to the federal level [9].

The reported cases of CS and the fetal and non-fetal deaths, whose underlying and/or associated cause was CS, were considered eligible. The death certificate, filled out by the assistant doctor, was the instrument used to collect data from SIM. Sinan uses data collected from standard forms for each case diagnosis that is defined in a national list [10] and provides information about morbidity in Brazil.

Eligible cases were infant and fetal deaths related to CS that were reported from January 1, 2007 through December 31, 2013 for SIM or from January 1, 2007 to September 30, 2013 for Sinan. (Period differences were related to updating of data bases during data collection). Cases were considered ineligible if they occurred outside the dates of the study interval, deaths were found upon review to be unrelated to CS, or if the deaths occurred outside the infant or fetal period. We used the Brazilian surveillance case definition of CS, that is, a live born infant, stillbirth or fetal loss born to a mother with serologic, microbiologic or clinical evidence of syphilis who was untreated or inadequately treated for syphilis at least 30 days prior to delivery [7]. Infants included babies born alive through 312 days of life.

The surveywas based on ICD-10 (International Classification of Diseases and Related Health Problems) codes ranging from A50.0 to A50.9 [11]. All cases of death from CS reported through SIM were used to estimate mortality rate and underreporting of deaths from CS.

Sinan and SIM databases were subjected to data quality assessment in order to detect, classify and remove duplicate records. In order toremove duplicate records from Sinan, we considered the first records (chronological order) and added complementary information from the subsequent records whenever needed.

We used the record linkage method, i.e., the integration of information from two independent sources, to minimize problems such as underreporting, and incomplete and duplicate data in both systems [12, 13].

Deterministic record linkage was performed using Microsoft Office Excel spreadsheets and common identifiers such as patient’s name, mother’s name, date of birth/expulsion of the conceptus, and date of death. Initially, database preprocessing was done to reformat variables (date of notification, date of diagnosis, and date of birth of the conceptus/child) and correct spelling errors in the variables (patient’s name, mother’s name, and phonetics).

The variables *mother’s name* and *phonetics* were used as a starting point for the identification of record matches/pairs through overlapping names registered in SIM and Sinan (identified by different colors) which were then placed in alphabetical order.We identified homonymous and similar names, and confirmed the matches through other information such as date of birth of the conceptus, mother’s age, State, municipality of residence, address, house number, and phone number. The variable *type of death* was used to divide datasets into smaller sets and separate fetal deaths from infant deaths to facilitate matching/pairing and analysis.

Cases presenting the same records (mother and children) regarding the same pregnancy were considered true matches, and the cases that were not found in both database were considered unpaired. The cases of death of children whose mother had a child with CS in previous pregnancies reported through SIM and not reported through Sinan were retained in datasets and considered multiple record pairs, as they mirror possible deaths from CS.

We assessed maternal variables (mother’s age; education; prenatal care; time of diagnosis; maternal Venereal Disease Research Laboratory (VDRL) titer; treatment of sexual partner; and maternal syphilis notifications in different pregnancies); and infant variables (presence of signs and/or symptoms, peripheral blood VDRL titer, age at death, case evolution/outcome, and classification of death into fetal death or infant death) from Sinan records.

Sinan variable *maternal treatment regimen*–classified as adequate/inadequate or not available–was not used for analysis after we analyzed and identified inconsistencies in the information on each case. This variable only provides the date of treatment and does not allow for the identification of the type of treatment and the number of doses given to the mother and sexual partner. Thus, due to inconsistencies in Sinan database regarding the classification of maternal treatment and differences between VDRL tests of mothers and children, the automatic classification of records to be discarded done by the Sinan data processing system was not considered. We excluded 43 cases whose data on classification of evolution/outcome were missing.

We defined the perinatal period to include fetal and infant deaths that occurred from 22 gestational weeks to the seventh day of life. The neonatal period covers the babies born alive who died within 28 days after birth, being the early period from zero a six days and the late period from seven to 28 days after birth [14]. To estimate the rates of stillbirths, perinatal mortality, and early and late neonatal mortality, we used data from the years 2007 to 2013 that were fully updated.

We estimated the proportion of underreporting of CS as a cause of fetal and infant death in the selected databases. We performed a descriptive analysis of the outcome of the conceptus and children with reported CS according to the study variables. Statistical significance was estimated using Pearson’s chi-squared test and Fisher’s test and set at p<0.05. The incidence rates of stillbirths and perinatal and neonatal mortality from CS were estimated for the years assessed in the present study. Data were tabulated and analyzed using the Statistical Package for the Social Sciences (SPSS) version 18.0.

The study was approved by the Research Ethics Committee of the University of Fortaleza (UNIFOR), being Opinion No. 072/2009. Since this is a study that uses secondary database whose information is collected routinely by professionals in health care during childbirth care, there was no need to request the signing of the consent form. Data were collected in the Mortality Information System and Notifiable Diseases Information System databases. An authorization term collection and use of data from information systems by the responsible department of the Health Department was signed.

The records of the study were kept private according to the stipulations of the law, therefore no mother or child identification could be divulged. This study is part of the research project titled “Evaluation of measures to prevent vertical transmission of syphilis in Fortaleza, Ceará” sponsored by FUNCAP/CNPq (Grant No. 700.460/2008).

## Results

The total of 5,222 death cases (3,827 infant deaths and 1,395 fetal deaths) whose mothers did not live in the city of Fortaleza, were excluded. After careful analysis of the observations contained in records of Sinan and its linkage to SIM, 11 records were corrected with regard to the classification of the CS case evolution, due to differences between databases. SIM records were chosen for the correction.

**Fig 1:**
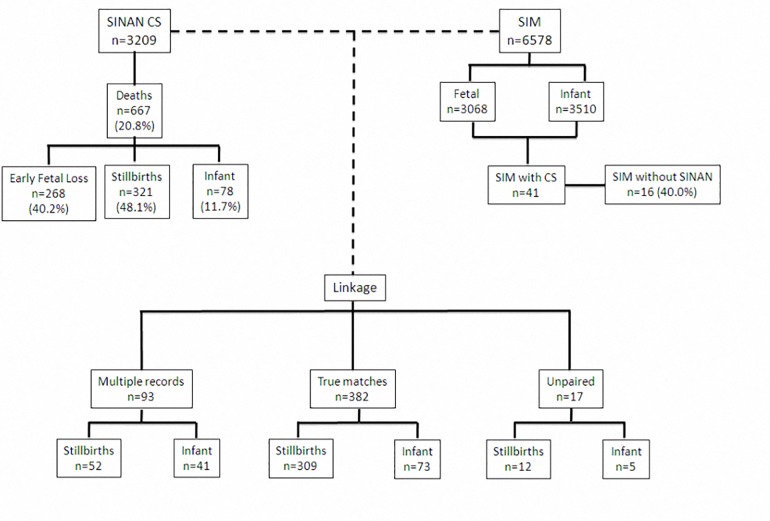
Deterministic record linkage between the *Sistema de Informação de Agravos de Notificação* (Sinan)-Congenital Syphilis and *Sistema de Informação sobre Mortalidade* (SIM) databases. Fortaleza, Northeastern, Brazil, 2007–2012.

In Sinan 3,209 cases of CS were identified, and 6,578 deaths were identified in SIM (3,068 fetal deaths and 3,510 infant deaths). After database linkage, we identified 382 cases that were reported in the SIM and Sinan databases, consisting of 309 (80.9%) fetal deaths and 73 (19.1%) infant deaths related to CS. The linkage rate between databases was 95.7%, excluded early fetal loss (Fig 1).

There were 93 multiple record pairs, we identified 52 (56%) possible stillbirths and 41 (44%) possible infant deaths from CS identified from mothers had conceptuses with reported CS in other pregnancies. There were five (6.4%) cases of newborn death and 12 (3.7%) stillbirths among unmatched records, which although were reported through Sinan, were not found in SIM. Of the 41 deaths reported through SIM as CS, 16 (39.0%) had not been reported into the syphilis surveillance system (Sinan) and were identified through this crossmatch linkage method (Fig 1).

Of the 3,209 CS cases reported through Sinan, 667 (20.8%) died. Of these, 268 (40.2%) were early fetal loss, 321 (48.1%) stillbirths and 78 (11.7%) infant deaths. Of the 399 fetal and infant deaths reported in Sinan, only 25 (6.3%) indicated CS as the cause of death in one of the lines of the death certificate (DC) (Fig 1). We identified underreporting of CS as a cause of death in 90% of the overall CS cases, with a stable rate across the time period studied (Table 1).

**Table 1:** Report of CS-associated fetal and infant death and extent of under-reporting of syphilis as a cause of CS death in SIM and Sinan. Fortaleza—Ceará, Brazil, 2007–2013.

Year	Syphilis reported as a cause of death through SIM^*^	Deaths from syphilis after analysis SIM/Sinan^*^	Proportion of underreporting of syphilis as cause of death
	(n)	(n)	(%)
2007	04	43	90.7
2008	03	48	93.8
2009	06	71	91.5
2010	08	72	88.9
2011	09	82	89.0
2012	04	65	93.8
2013^**^	07	34	79.4
Total	41	415	90.1

*Syphilis reported as one of the causes of death

**Analysis until September 2013

From the 2,542 children that born alive notified at Sinan database, 78 (3.1%) died; Out of these, 39 (50.0%) were early neonatal deaths and 25 (32.1%) were late neonatal deaths. In 14 (17.9%) records, the information about age at death was missing.

**Fig 2:**
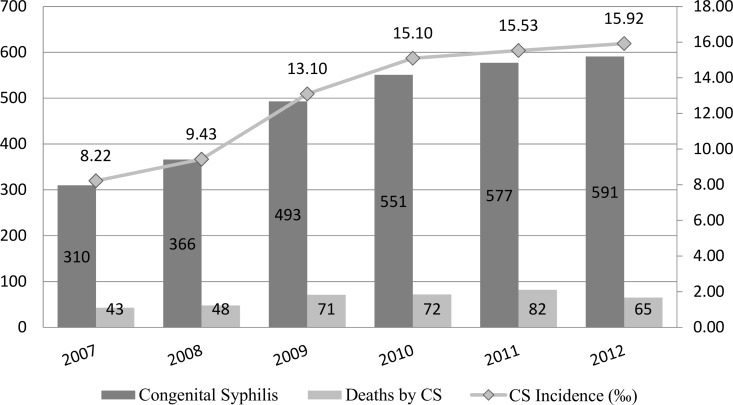
Congenital Syphilis Incidence Rates per 1000 Live births and Congenital Syphilis, by Year of Notification, Fortaleza-Ceará, 2007–2012. Source: Secretaria de Saúde de Fortaleza, Sistema Nacional de Agravos de Notificação (Sinan).

Fig 2 shows CS incidence rate in Fortaleza city by year of diagnosis. There has been a rise in incidence rates over time, with an increase of circa 90.6% between 2007 and 2012. The number of cases increased an average of 14.3% per year, with an increase of 34.7% between 2008 and 2009. The highest incidence rates were recorded in 2011 and 2012, with 15.5 and 15.9 cases per 1,000 live births (LB), respectively.

**Fig 3:**
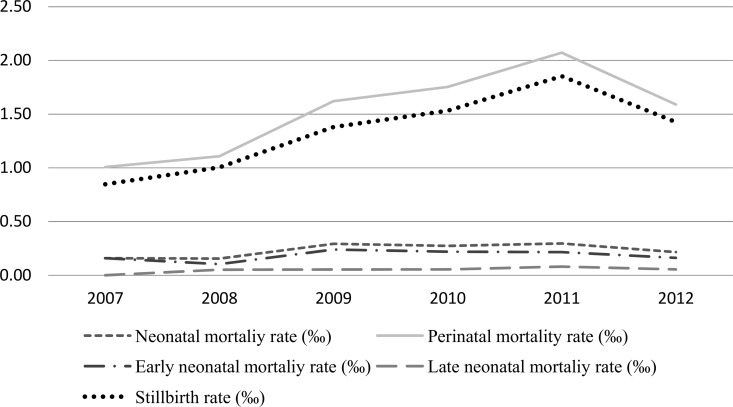
Rates of Stillbirth, Perinatal and Neonatal Mortality of Congenital Syphilis cases from 2007 to 2012. **Fortaleza—Ceará, 2007–2012.** Source: Fortaleza Heath Secretary. Mortality Information System (SIM).

CS was associated to 373 perinatal deaths between 2007 and 2012, with a perinatal mortality rate of 1.66 per 1,000 LB and a stillbirth rate of 1.34 per 1,000 LB. These rates presented an increasing trend over the years, however, in 2012 there was a decline in comparison to 2011 (-0.48 to -0.42). The same situation has not been observed in the neonatal mortality rate, which remained stable during the same period. We found that CS was responsible for a higher proportion of perinatal deaths than neonatal deaths, and early neonatal mortality was higher than late neonatal mortality (Fig 3).

Table 2 shows the analysis of factors associated with death of conceptus infected with CS. The following variables were significantly associated with fetal and infant death outcomes: mother’s age ≥19 years (p = 0.02); mother who did not attend prenatal care (p<0.001); mother tested for syphilis at the time of delivery/curettage or not tested (p<0.001); maternal titer at the time of delivery/curettage higher than 1:8 (p<0.001); untreated sexual partner (p<0.001); peripheral blood titer of newborns (NB) higher than 1:8 (p = 0.002); and children presenting signs or symptoms (prematurity, low birth weight, anemia, jaundice, respiratory distress, visceromegaly, congenital malformations, serosanguinous rhinitis, skin lesions, heart diseases and/or hearing loss, among others) (p<0.001).

**Table 2:** Maternal and other factors associated with CS death or CS live infant, Fortaleza–Ceará, 2007–2013.

Variables	Live		Nonlive		
	n	%	n	%	p value
Mother’s age (n = 3,142)					0.025
≤ 19 years	611	76.3	190	23.7	
≥ 20 years	1873	80.0	468	20.0	
Mother’s education (n = 2,415)					0.352
Up to 9 years	1675	88.4	219	11.6	
> 9 years	453	86.9	68	13.1	
Attended prenatal care (n = 3,071)					<0.001
Yes	1872	89.7	215	10.3	
No	624	63.4	360	36.6	
Syphilis diagnosis (n = 3,078)					<0.001
During pregnancy	1193	94.2	74	5.8	
At the time of delivery/curettage/not tested	1255	69.3	556	30.7	
Maternal titer at the time of delivery (n = 3,095)					<0.001
≤ 1:8	1562	86.4	245	13.6	
> 1:8	876	68.0	412	32.0	
Partner received treatment (n = 3,209)					<0.001
Yes	417	95.2	21	4.8	
No/Missing	2125	76.7	646	23.3	
Peripheral blood titer of NB^*^(n = 1,914)					0.002
≤ 1:8	1568	98.3	27	1.7	
> 1:8	304	95.3	15	4.7	
Presence of signs or symptoms in NB^*^(n = 2,020)					<0.001
Yes	487	91.0	48	9.0	
No	1465	98.7	20	1.3	

* Newborn who were born alive and then died

Source: Sistema Nacional de Agravos de Notificação (Sinan).

In the multivariate analysis, the only variable that maintained statistical significance for the death of the conceptus and children was the appearance of CS signs and / or symptoms at birth (OR = 3.20; IC95% 1.54–6.62; p = 0.002) (Table 3).

**Table 3:** Pregnancy outcome after adjusted and not adjusted multivariable logistic regression analysis. Fortaleza—Ceará, 2007–2013.

	Pregnancy Outcome							
Not alive								
Variables	Not adjusted					Adjusted		
	n/N	%	OR	(IC-95%)	p value	OR	(IC-95%)	p value
Mother age					0.025	1.83	(.90–3.70)	0.09
≤ 19	190/658	28.9	1					
> 19	611/2484	24.6	1.24	(1.02–1.51)				
Prenatal					<0.001	1.07	(0.45–2.52)	0.87
Yes	360/575	62.6	5.02					
No	624/2496	25.0	1	(4.12–6.11)				
Mother syphilis diagnoses					<0.001	1.49	(0.60–3.66)	0.37
During prenatal care	74/630	11.7	7.14					
At labor	1255/2448	51.3	1	(5.51–9.34)				
Treated partner					<0.001	1.89	(0.42–8.37)	0.40
Yes	2125	83.6	6.03					
No	646/154	96.8	1	(2.49–3.59)				
Mother VDRL result at labor					<0.001	1.55	(0.68–3.50)	0.29
≤ 1:8	876	35.9	2.99					
> 1:8	412/657	62.7	1	(1.17–4.36)				
Newborn peripheral blood VDRL result					0.001	1.58	(0.68–3.67)	0.28
≤ 1:8	304/1872	16.2	2.86					
> 1:8	15/42	37.1	1	(1.39–5.65)				
Newborn clinic diagnoses					<0.001	3.20	(1.54–6.62)	0.002
Symptomatic	48/68	70.1	1					
Asymptomatic	487/1952	24.9	7.21	(4.15–12.9)				

## Discussion

In Brazil, the poor quality and underreporting of health data greatly compromise the analysis of deaths, especially with regard to fetal and early neonatal deaths [14,15]. The present study found a high proportion of underreporting of fetal and neonatal deaths due to CS in both information systems (SIM and Sinan). The recording and analysis of these data allowed linkage and are important to the recognition of CS as a problem that needs to be addressed effectively.

In the present study, the application of the record linkage method allowed to link CS data and information on CS-related mortality in 95.7% of cases, which is similar to other studies that have used the same technique [12,13]. A recent study in the state of Amazonas also found significant underreporting of CS as a cause of fetal or infant death. That study also found CS-related mortality that has probably been caused by this disease in the city [15].

Underreporting appears to be related to certain aspects of the reporting systems. For example, CS is not listed as a possible choice for underlying cause of death, and rather must be recorded on one of the lines of the Death Record. SIM follows the basic rules of death classification established by WHO, prioritizing some causes of death as attributed to CS or CS as a contributor to the cause of death.

The lack of recognition and visibility of CS as a major public health problem may also be associated with its non-classification as a cause of fetal and infant death, especially fetal. Saraceni et al [4] have suggested the monitoring of perinatal mortality from CS as a strategy for the disease control, and WHO has expanded this recommendation to fetal mortality in 2010 [16]. Underreporting of fetal and neonatal death should be minimized through careful analysis of data on fetal and infant deaths by Investigation Committees. These committees should consider CS as one of the causes of death if the mother or child have been diagnosed with syphilis, even if other causes that possibly led to the child's death are included in the Death Record.

As is true in Brazil overall, CS incidence rates in the city of Fortaleza have increased over recent years [7]. Recent CS increases may be related to a country-wide stock out of long-acting penicillin regimens in primary health care settings. Intramuscular injection of long acting penicillins (e.g., benzathine benzyl penicillin) is the treatment recommended for syphilis regardless of stage. Sexually acquired syphilis in adults can be treated with oral, non-penicillin regimens; however, because these drugs do not reach the fetus, only injectable penicillin is effective in treating syphilis in pregnancy. Recent shortages of benzathine penicillin reduce the chance of effective treatment of syphilis-infected pregnant women and their sexual partners, thus increase the number of CS cases. Lack of effective treatment (penicillin) may also lead to more syphilis in the community, increasing the chances that pregnant women are infected and leading to more CS.

The increase in CS deaths, particularly those occurring during the perinatal period (97,6% of deaths identified), may also be related to poor coverage or quality of prenatal care. The critical importance of early prenatal care that promotes early screening and treatment of syphilis, ideally in the first trimester of pregnancy, cannot be over emphasized. CS is responsible for many adverse pregnancy outcomes including prematurity, hydrops fetal, and intrauterine growth restriction, all of which increase perinatal mortality [17].

This study was also important in identifying missed clinical opportunities for CS treatment as well as underreporting of CS. Over 32% of children died after hospital discharge, a situation that may be related to the lack of treatment at the maternity hospital or the inadequate follow-up. Negative results of the VDRL in peripheral blood at delivery may turn positive later on. However, these results are widely considered in medical treatment decisions, and children may be discharged without proper management and follow-up after hospital discharge [18].

This study was also important in identifying missed clinical opportunities for CS treatment as well as underreporting of CS. Over 32% of children died. Lower prenatal care attendance was associated with fetal or infant death outcomes as well as the high proportion of fetal mortality among mothers who were only tested at the time of delivery. This reinforce the importance of improving the quality of prenatal care for the prevention of CS cases, a situation that has also been demonstrated by other studies [19,20]. A study to estimate the incidence of CS in Brazil and its relationship to the coverage of the Family Health Strategy found that despite the increase in prenatal care coverage, the CS prevention actions in the country are minimally effective [21] once the CS vertical trnsmission prevention were not properly made. Other studies have also found low syphilis testing coverage in prenatal care, often reducing the chances of detecting the reinfection of pregnant women after treatment and the infection in more advanced stages of pregnancy [3,22].

The Family Health Strategy plays an important role in the reduction of vertical transmission of syphilis, as the effectiveness of CS control actions and the low cost of their implementation have been proven in many studies [18,23,24]. The Family Health Strategy aims to reorganize primary care in Brazil and is seen by the Brazilian Health Ministry as expansion, qualification and consolidation strategy of primary care for favoring the principles, guidelines and fundamentals of primary care, that is to enlarge the solvability and impact on people health situation. Moreover, the relationship and proximity to the community as well as the easy access to pregnant women enable early serology testing and the development of mechanisms and strategies for the adequate treatment of pregnant women and their sexual partners [25].

This study has some limitations. One major limitation was that the analysis used secondary data, which can be fraught with inconsistences that limit a more accurate and detailed analysis of some variables. Some data may have been missing, limiting our ability to identify the maternal or other factors most closely associated with CS death. Additionally, typographical errors may have contributed to the loss of some cases during linking. Although we used several means of limiting these inaccuracies, we could not limit all problems. As we did not integrate data on cases of pregnant women with syphilis reported through SIM and Sinan, some cases of death from CS may have been underestimated. However, data on cases of pregnant women reported with syphilis are very limited and inferior to data on CS. Moreover, the fact that mothers are infected with syphilis does not necessarily mean that the child died from CS, although the disease morbidity and mortality are well known.

The underreporting of CS as one of the causes of fetal and infant death leads to unawareness of the reality of deaths from this disease. This situation has major consequences, as CS remains little known by health managers, hindering the development of public policies aimed at its prevention.
